# The influence of fractionated radiotherapy on the stability of spinal bone metastases: a retrospective analysis from 1047 cases

**DOI:** 10.1186/s13014-018-1082-2

**Published:** 2018-07-24

**Authors:** Tanja Sprave, Katharina Hees, Thomas Bruckner, Robert Foerster, Tilman Bostel, Ingmar Schlampp, Rami El Shafie, Nils Henrik Nicolay, Juergen Debus, Harald Rief

**Affiliations:** 1 Department of Radiation Oncology, University Hospital Heidelberg, Im Neuenheimer Feld 400, 69120 Heidelberg, Germany; 2 Department of Medical Biometry, University Hospital Heidelberg, Im Neuenheimer Feld 130.3, 69120 Heidelberg, Germany; 3 National Center for Radiation Research in Oncology (NCRO), Heidelberg Institute for Radiation Oncology (HIRO), Heidelberg, Germany; 4 Department of Radiation Oncology, University Hospital Zurich, Raemistrasse 100, 8091 Zurich, Switzerland; 5 German Cancer Research Center (DKFZ), Clinical Cooperation Unit Radiation Oncology, Im Neuenheimer Feld 280, 69120 Heidelberg, Germany

**Keywords:** Bone metastases, Stability, Fractionation, Palliative radiotherapy

## Abstract

**Background:**

The effect of radiotherapy, in particular the application of different multi-fraction schedules in the management of unstable spinal bone metastases (SBM), is incompletely understood. This study aims to compare the radiological response regarding various dose and fractionation schedules of radiotherapy in the palliative treatment of SBM.

**Methods:**

We retrospectively assessed 1047 patients with osteolytic SBM, treated with palliative radiotherapy at our department between 2000 and 2015. Lung cancer (40.2%), breast (16.7%) and renal cancer (15.2%) were the most common solid tumors in this study. Different common multi-fraction regimen (5x4Gy, 10x3Gy, 14 × 2.5Gy and 20x2Gy) were compared with regard to radiological response and recalcification at 3 and 6 months after radiotherapy. The Taneichi score was used for classification of osteolytic SBM.

**Results:**

Median follow up was 6.3 months. The median overall survival (OS) in the short-course radiotherapy (SCR) group using less than 10 treatment fractions was 5.5 months vs. 9.5 months in the long-course radiotherapy (LCR) group using in excess of 10 fractions (log rank *p* < .0001). Overall survival (OS) in the SCR group after 3 and 6 months was 66.8 and 49.1%, respectively vs 80.9 and 61.5%, respectively in the LCR group.

17.6% (*n* = 54/306) and 31.1% (*n* = 89/286) of unstable SBM were classified as stable in the SCR group at 3 and 6 months post radiotherapy, respectively (*p* < .001 for both). In the LCR group, 24.1% (*n* = 28/116) and 34.2% (*n* = 38/111) of unstable SBM were stabilized after 3 and 6 months, respectively (*p* < .001 for both).

**Conclusions:**

Our study shows no significant difference in stabilization achieving recalcification rates between multi-fraction schedules (SCR vs. LCR) in the palliative management of unstable SBM. Both groups with multi-fraction regimen demonstrate a stabilizing effect following 3 and 6 months after radiotherapy.

## Background

Spinal bone metastases occur in up to 40% of tumor patients during the course of the disease [1].

Associated spine pain, immobility, pathological fractures and neurological deficits substantially reduce the quality of life. Unstable SBMs in particular require coordinated multimodal therapy. In the palliative multidisciplinary approach, surgical intervention of the cervical and thoracolumbar spinal instability can achieve rapidly improved functional results [2–4]. Less-invasive surgery followed by early adjuvant radiotherapy could be a promising, safe and effective treatment option for achieving solid and durable stability in selected patients without metastatic spinal cord compression (MSCC) [3].

If surgical intervention is not possible, external beam radiotherapy (EBRT) is an established treatment for stabilization in the palliative management of patients with unstable SBM [5–11]. Numerous studies have investigated the influence of total dose and fractionation regarding to the pain response [12–23].

However, the optimal total dosage and fractionation in relation to the re-ossification of the unstable SBMs is still unclear. Chow and colleges did not find a significant difference in bone density between 8 Gy in a single fraction and 20 Gy in 5 fractions or 30 Gy in 10 fractions in patients with breast cancer 3 months follow up after irradiation [5]. Reinhold et al. described an increase in bone density over 60% of lytic vertebral metastases at 3 months after 40 Gy in 20 fractions in subgroup with 13 participants [6].

We initiated this retrospective mono-centric analysis to investigate the influence of different fractionation schemes and doses on the re-ossification rate in metastatic solid cancers. To the best of our knowledge, no comparable study has been described in the literature so far.

## Methods

### Assessment of the radiological response

From 2000 to 2015, 1047 patients with histologically confirmed solid tumors and osteolytic SBM were treated with external beam RT (EBRT) at our department. Patient data were taken from the cancer registry of the National Center for Tumor Diseases (Heidelberg, Germany). SBM were diagnosed using computed tomography (CT) and magnetic resonance imaging (MRI).

The stability of osteolytic SBM was examined according to the Taneichi score [24] based on the CT imaging before EBRT and at 3 and 6 months after treatment. This score classified osteolytic SBM as “stable” or “unstable” by definition of risk factors such as tumor size and the degree of costovertebral joint destruction for the thoracic region (Th 1 to 10) and tumor size and the degree of pedicle destruction for the lumbar segments (Th 11 to L5). Osteolytic metastases were rated on a scale from A to G, whereby subtypes A to C were defined as stable, and subtypes D to G as unstable. In cases with multiple metastases in which only one lesion was detected as unstable, the Taneichi score was defined as unstable.

After 3 and 6 months, 621 and 590 cases were evaluated in the SCR group vs. 205 and 197 cases in the LCR group, respectively. The initial classification of the osseous metastases into stable or unstable was made by experienced radiologists based on CT imaging. Subsequent evaluation was performed after 3 and 6 months using the baseline imaging, the changes in bone density, and the extent of the stabilizing resclerosing of the osteolytic lesions.

Bone density in irradiated vertebral bodies was assessed at baseline and at 3 and 6 months after RT. Bone density was assessed with the Syngo Osteo CT workstation in manually selected regions of interest (ROIs). Hounsfield units (HU) were used for bone density measurements. Siemens Somatom Sensation Open (Siemens, Erlangen, Germany) was used for all CT examinations. Measurements were carried out at the appropriate site by a single physician.

This study was approved by the independent ethics committee of the Heidelberg Ethics Committee on 22 October 2012 (# S-513/2012). Due the retrospective design, informed consent was not required. This retrospective study analyzes/utilizes the cumulative patient data taken from previously published studies, which also evaluated the influence of multi-fraction radiotherapy on the stability of osteolytic SBM, but employed entirely different aspects [7, 10, 25, 26].

### Statistical analysis

The empirical distribution of continuous variables is described by the number of observations, mean and standard deviation; scores were described by median and range, the description of categorical variables includes the number and percentage of patients belonging to the relevant categories. Possible differences between the group of patients with <= 10 fractions compared to those with > 10 fractions were evaluated with t-test for continuous data, with Mann-Whitney U-test for ordinal data (scores) and with chi-square-test for categorical data. Multivariate analysis was performed using binary logistic regression. This analysis evaluates possible prognostic factors of baseline stability, namely age, gender, KPS, primary site, localization and number and type of metastasis, calculated separately for patients with SCR or LCR.

“Bone survival” (BS) was defined as the time from initial diagnosis of SBM until death. The time of site irradiation was not equal to the time of initial diagnosis of SBM. Overall survival (OS) was defined as time from the beginning of RT until death. We estimated patient survival using the Kaplan–Meier method. Patients were censored on the basis of whether or not they were alive. Possible differences were reported with *p*-values of the log-rank tests. All statistical analyses were done using the SAS software version 9.4 (SAS Institute, Cary, NC, USA).

### Patient’s characteristics

Palliative EBRT was required for analgesia in 53.9% (*n* = 564), stabilizing intention in 42.6% (*n* = 446) and for neurological deficits in 2.2% (*n* = 23). Only 14 patients (1,3%) had adjuvant irradiation after prior surgical intervention.

Thus, 534 cases (51%) were classified as unstable SBM, and a stabilizing orthopedic corset was indicated in 475 cases (45.4%). Because the Taneichi score application is limited to thoracic and lumbar spine segments, in this study the most SBM were consequently localized in 60.7% (*n* = 635), in the thoracic spine and in lumbar spine 39.2% (*n* = 410).

Patients’ mean age at diagnosis of SBM was 63.1 years (SD +/− 11.1 years), and median Karnofsky performance status (KPS) was 80.0% (range 30–100) [27]. Gender was well balanced with 578 male (55.2%) and 469 female patients (44.8%).The most frequent tumor type was non-small cell lung cancer (NSCLC) with 40.6% (*n* = 425), followed by breast cancer 16.7% (*n* = 175) and renal cancer 15.2% (*n* = 159). Antiresoptive treatment such as bisphosphonate or denosumab was received by 62.6% participants. The characteristics of all participants included in this analysis are summarized in Table 1.

**Table 1:** Demographics

	<= 10 fractions		> 10 fractions		All patients		*p*-value
	*n* = 769		*n* = 278		*n* = 1047		
	*n*	%	*n*	%	*n*	%	
Age (years)							
Mean (SD)	63.3 +/− 10.7		62.5 +/− 12.0		63.1 +/− 11.1		0.330^a^
Gender							
Male	435	56.6	143	51.4	578	55.2	0.141^b^
Female	334	43.4	136	48.6	469	44.8	
Karnofsky-index							
Median (range)	80 (40–100)		80 (30–100)		80 (30–100)		0.696^c^
Primary site							< 0.001^b^
NSCLC	356	46.3	69	24.8	425	40.6	
Breast cancer	106	13.8	69	24.8	175	16.7	
Renal cancer	88	11.4	71	25.5	159	15.2	
Other	219	28.5	69	24.8	288	27.5	
Localization of metastases							0.476^b^
Cervical	1	0.1	1	0.3	2	0.2	
Thoracic	460	59.8	145	63.0	635	60.7	
Lumbar	308	40.1	102	36.7	410	39.1	
Number of metastases							0.329^b^
Mean (SD)	2.6 +/− 2.1		2.9 +/− 2.8		2.7 +/− 2.3		0.069^a^
Solitary	350	45.5	136	48.9	486	46.4	
Multiple	419	54.5	142	51.1	561	53.6	
Other distant metastases							
Liver	203	26.4	60	21.6	263	25.1	0.112^b^
Brain	112	14.6	30	10.8	142	13.6	0.119^b^
Lung	185	24.1	74	26.6	259	24.7	0.396^b^
Tissue	55	7.2	12	4.3	67	6.4	0.097^b^
Antiresoptive treatment	513	66.7	142	51.1	655	62.6	< 0.001^b^
Chemotherapy	445	57.9	145	52.4	590	56.4	0.112^b^
Stable metastases	392	51.0	121	43.5	513	49.0	0.033^b^
Unstable metastases	377	49.0	157	56.5	534	51.0	
Orthopedic corset	353	45.9	122	43.9	475	45.4	0.562^b^
Radiotherapy schedule							
1 × 8 Gy					1	0.1	
5 × 4 Gy					11	1.0	
10 × 3 Gy					757	72.3	
11 × 3 Gy					1	0.1	
12 × 3 Gy					1	0.1	
14 × 2.5 Gy					92	8.8	
20 × 2 Gy					184	17.6	

Baseline characteristics of participants. Explanation: Others: colon cancer, neuroendocrine cancer, prostata cancer, ovarian cancer, urothelial cancer, uterine cancer, vulva cancer. Abbreviation: *NSCLC* non-small-cell lung carcinoma; ^a^t-test; ^b^chi-square-test, ^c^u-test

### Radiotherapy

After virtual simulation, RT was performed as irradiation of the involved vertebral body as well as the vertebrae immediately above and below using 6 MV individually-formed beams after CT-scan based 3D-planning. Palliative radiotherapy was delivered in most cases with 10 × 3 Gy (72.3%; *n* = 757), 20 × 2 Gy (17.6%; *n* = 184), 14 × 2.5 Gy (8.8%; *n* = 92) and other individual doses (1.4%; n = 15). The therapy schedule was prescribed according to general performance status, histology of primary solid tumor, prognosis and life expectancy.

Two multi-fraction groups was formed: patients who received shot course radiotherapy (SCR) with </=10 fractions vs. patients undergoing long course radiotherapy (LCR) > 10 fraction.

## Results

Median follow-up time for both groups was 6.3 months (range 0.03–283 months).

Initially, 51% (*n* = 534) of 1047 SBM were classified as unstable SBM, of which 377 were in the SCR and 157 in the LCR group.

After 3 months, 621 with SCR and 205 patients with LCR could be re-examined, the originally unstable SBM were reclassified as stable in 17.6% (54/306) of the cases for the SCR group vs. 24.1% (28/116) for the LCR group (McNemar’s test *p* < .001). After 6 months, 590 with SCR and 197 patients with LCR could be re-examined, and 31.1% of initially unstable SBM (89/286) were classified as stable in the SCR group vs 34.2% (38/111) for the LCS group. The frequent reasons for lost of follow up were worsening of the condition, continuation of therapy in other clinics or death.

Conversely, in the SCR group at 3 and 6 months follow RT 1.6% (5/315) and 2.0% (6/304) of the stable SBM were reclassified as unstable. A numerically comparable destabilizing rate was also found in the LCR group after 3 and 6 months respectively 4.5% (4/89) and 7% (6/86) (Table 2).

**Table 2:** Stability after 3 and 6 months in both groups

<= 10 fractions		3 months				
	Unstable		Stable		Total	
	*n*	%	*n*	%	*n*	%
Unstable	252	82.4	54	17.6	306	49.3
Stable	5	1.6	310	98.4	315	50.7
					621	100
> 10 fractions						
Unstable	88	75.9	28	24.1	116	56.6
Stable	4	4.5	85	95.5	89	43.4
					205	100
<= 10 fractions		6 months				
	Unstable		Stable		Total	
	*n*	%	*n*	%	*n*	%
	197	68.9	89	31.1	286	48.5
	6	2.0	298	98.0	304	51.5
					590	100
> 10 fractions						
Unstable	73	65.8	38	34.2	111	56.4
Stable	6	7.0	80	93.0	86	43.7
					197	100

**Fig. 1:**
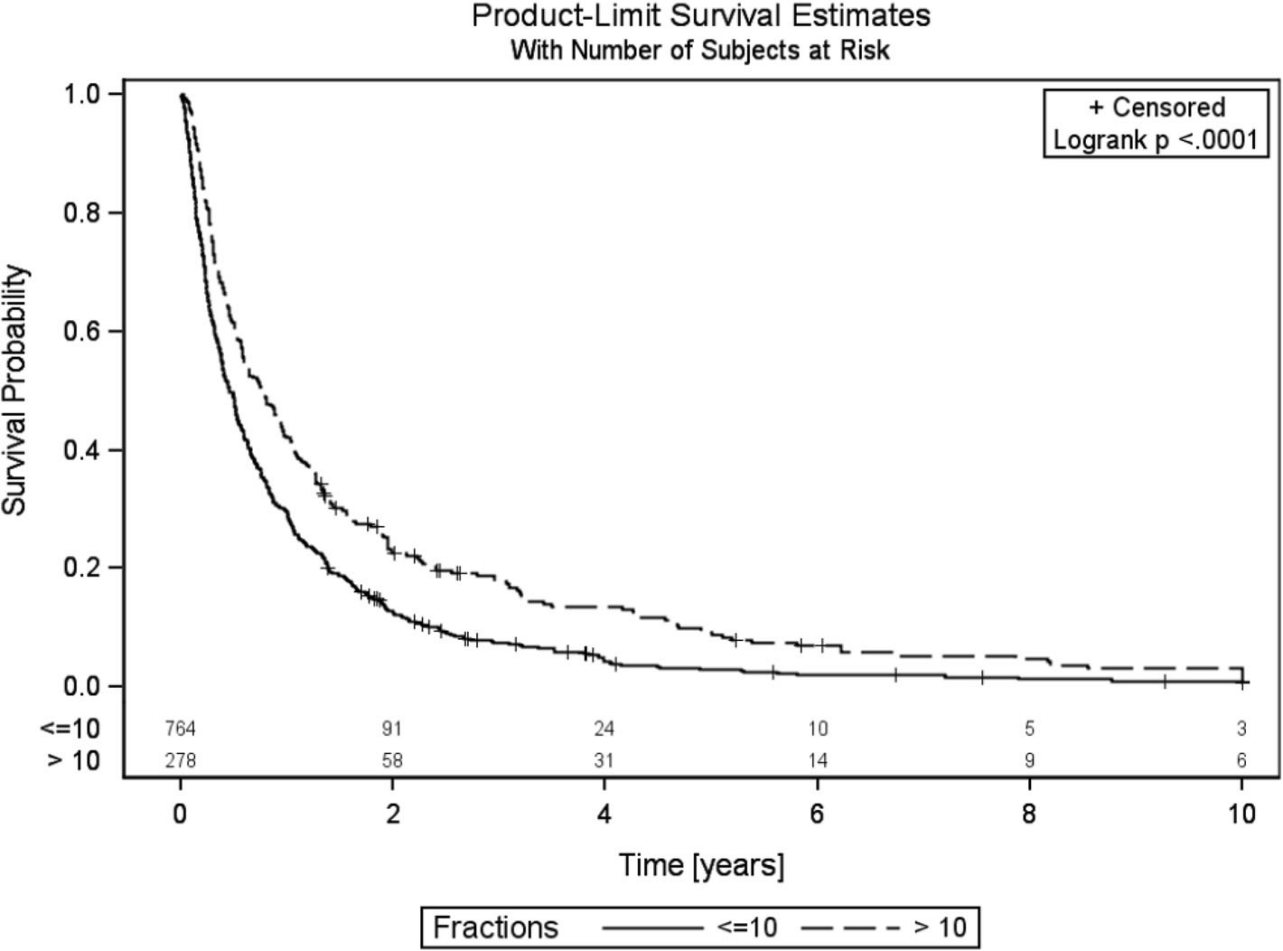
Overall survival of both arms, time in years

The median overall survival (OS) in the SCR group was 5.5 months vs. 9.5 months in the LCR (log rank *p* < .0001). The OS in the SCR group after 3 and 6 months was 66.8 and 49.1%, respectively vs 80.9 and 61.5%, respectively in the LCR group (Fig. 1). The stability dependent OS analysis showed no significant difference either for both groups or separately (log rank test for both groups: *p* = 0.631, SCR: *p* = 0.181 and LCR *p* = 0.946). At the last follow-up, 3.8% (*n* = 26) patients were still alive in the SCR group vs. 6.1% (*n* = 17) in the LCR group (chi square test *p* = 0.049).

**Fig. 2:**
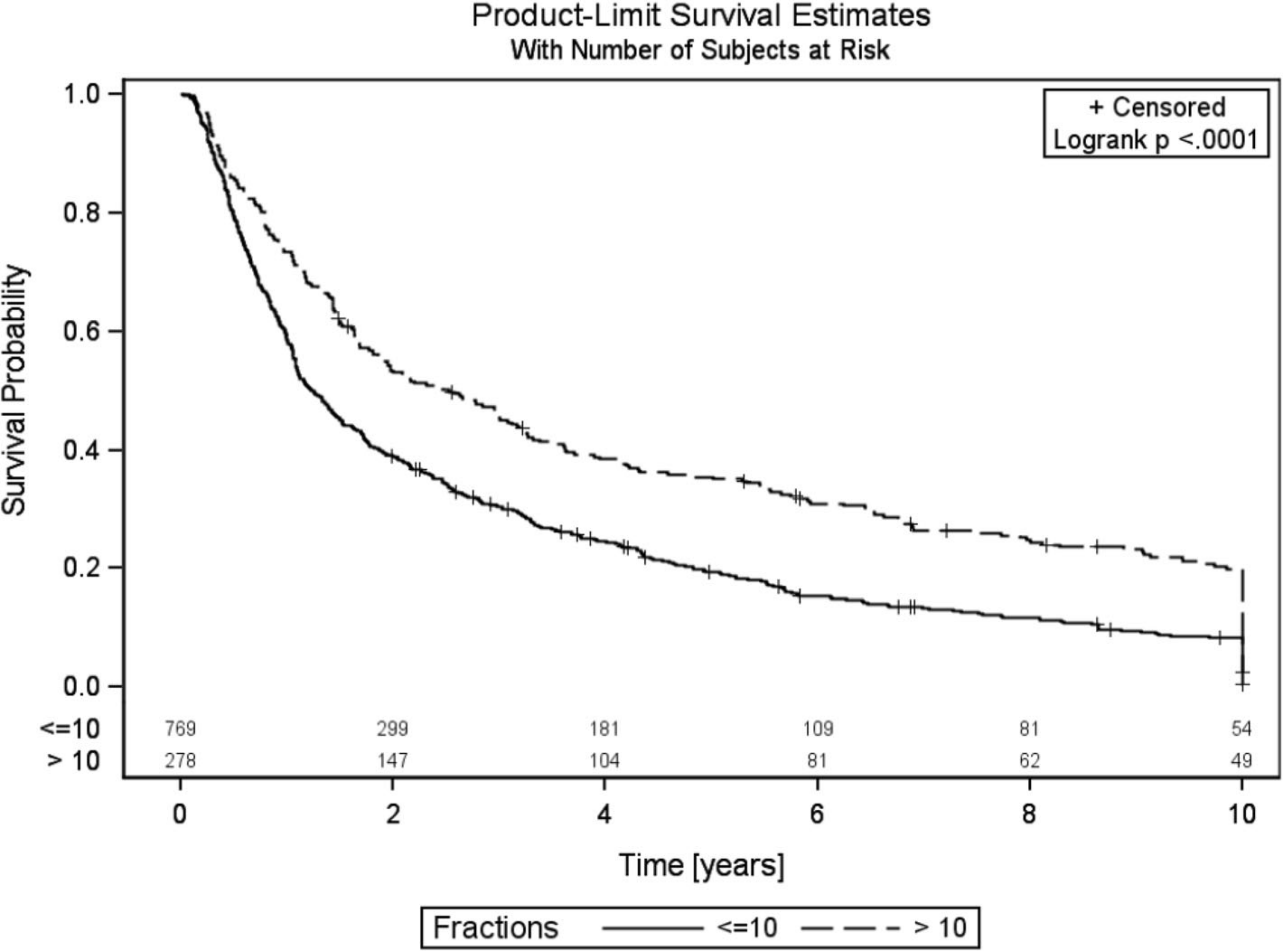
Bone survival of both arms, time in years

The median bone survival (BS) in SCR group was 6.7 months vs. 12.2 months in the LCR group (log rank *p* < .0001). (Fig. 2).

Gender had no influence on stabilization rate in both groups. SCR was delivered in 769 cases (73.4%) vs. LCR in 278 cases (26.6%). Antiresoptive treatment was prescribed to 62.6% of all patients, 66.7% in the SCR collective vs. 51.1% in the LCR group (chi square test *p* < 0.001).

The univariate analysis within the SCR group showed a significant difference in KPS between stable and unstable SBM (Mann-Whitney U-test, *p* = 0.027). Multivariate analysis in the SCR group showed a significant association between multiple metastases and instability (logistic regression, *p* < 0.0001) (Table 3).

**Table 3:** Univariate and multivariate analysis in both groups

	<= 10 fractions					
	stable		instable		*p*-value	*p*-value (logistic Regression)
	*n* = 377		*n* = 392			
	*n*	%	*n*	%		
Age (years)						
Mean (SD)	62.6 +/− 10.4		64.0 +/− 11.0		0.084^a^	0.214
Gender						
Male	219	55.9	216	57.3	0.690^a^	0.742
Female	173	44.1	161	42.7		
Karnofsky- Index						
Median (range)	80 (40,100)		70 (40,100)		0.027^c^	0.090
Primary site					0.933^b^	0.883
NSCLC	183	46.7	173	45.9		
Breast cancer	55	14.0	51	13.5		
Renal cancer	42	10.7	46	12.2		
Other	112	28.6	107	28.4		
Localization metastases					0.303^b^	0.637
Cervical/Thoracic	228	58.2	233	61.8		
Lumbar	164	41.8	144	38.2		
Number metastases					< 0.0001^b^	< 0.0001
Solitary	210	53.6	140	37.1		
Multiple	182	46.4	237	62.9		
Other distant metastases						
Liver	102	26.0	101	26.8	0.809^b^	0.518
Brain	59	15.1	53	14.1	0.697^b^	0.759
Lung	95	24.2	90	23.9	0.907^b^	0.902
Tissue	35	8.9	20	5.3	0.051^b^	0.120
	> 10 fractions					
	stable		instable		*p*-value	*p*-value (log. Regression)
	*n* = 121		*n* = 157			
	*n*	%	*n*	%		
Age (years)						
Mean (SD)	60.7 +/− 12.7		63.8 +/− 11.3		0.034^a^	0.168
Gender						
Male	60	49.6	83	52.9	0.588^a^	0.643
Female	61	50.4	74	47.1		
Karnofsky- Index						
Median (range)	80 (40,100)		70 (30,100)		0.063^c^	0.079
Primary site					0.575^b^	0.933
NSCLC	28	23.1	41	26.1		
Breast cancer	28	23.1	41	26.1		
Renal cancer	30	24.8	41	26.1		
Other	35	28.9	34	21.7		
Localization metastases					0.687^b^	0.970
Cervical/Thoracic	75	62.0	101	64.3		
Lumbar	46	38.0	56	35.7		
Number metastases					0.662^b^	0.834
Solitary	61	50.4	75	47.8		
Multiple	60	49.6	82	52.2		
Other distant metastases						
Liver	32	26.6	28	17.8	0.084^b^	0.965
Brain	17	14.1	13	8.3	0.129^b^	0.274
Lung	45	37.2	29	18.5	< 0.001^b^	0.013
Tissue	8	6.6	4	2.6	0.098^b^	0.406

Abbreviation: ^a^t-test; ^b^chi-square-test, ^c^u-test

The univariate analysis within the LCR group found a significant relationship between age and unstable SBM (Mann-Whitney U-test, *p* = 0.034). Multivariate analysis in this group showed a significant association between pulmonary metastases and instability (logistic regression, *p* < 0.013) (Table 3).

## Discussion

This retrospective study compared radiological response by various dose and fractionation schedules of radiotherapy in the palliative treatment of SBM. All multi-fraction schedules showed a stabilizing effect, and there was stabilization observed both in the SCR and LCR groups. We determined a statistically significant stabilizing effect after 3 and 6 months within each group (*p* < .0001). There was no difference between the groups.

There are few relevant studies that investigate the impact of fractionation on remineralization, but not stabilization of lytic bone lesions. The available data show a great heterogeneity of the primary endpoints and patient collective. Therefore, our results are difficult to compare with other studies.

The reossification of osteolysis as a surrogate for radiotherapeutic response has been widely investigated and integrated into daily routine. However, the relevant studies show very contradictory results. A prospective study by Chow et al. was conducted to examine the feasibility of the evaluation based on CT exams, the remineralization of the osteolytic bone lesions at 3 months after palliative radiotherapy. Radiotherapy was applied with 8 Gy in a single fraction vs. 20 Gy in 5 fractions or 30 Gy in 10 fractions. Of 25 breast cancer participants, 11 patients suffered from osteolytic vertebral bone metastasis. Bisphosphonate therapy was allowed. At the 3 months follow up no significant change of the median % bone density was assessed. There was no further description of the lytic metastases with regard to stability prior and after irradiation [5].

In contrast, Koswig and Budach showed a significant difference in recalcification of the osteolytic bone metastasis in favor of the fractionated group in a randomized two-arm (1 × 8 Gy vs. 10 × 3 Gy) trial. In the multi-fraction group, a significant effect was assessed only in patients with breast cancer (*p* < 0.001). No information on stability was provided [8].

In line with above mentioned previous study, Wachenfeld et al. assessed in 14 patients with vertebral bone lesions of breast cancer different radiological response of the irradiated bone varies depending on the form of metastasis. Accordingly, osteolytic metastases generally showed an increase in CT density to approximately 150% of the initial value at 3 months after the multi-fraction irradiation. This effect was further enhanced by additional chemotherapy [28]. Two decades later, we showed similar results in a retrospective analysis of 115 patients with 135 spinal metastases from the breast cancer. In contrast, no correlation between differences in the bone density and simultaneous chemotherapy was observed [7].

Notably, different fractionation showed no difference in the recalcification rates of multiple myeloma bone lesions [29]. The randomized trial by Rudzianskiene et al. was conducted to compare two schedules (1 × 8 Gy vs. 10 × 3 Gy) in palliative treatment of multiple myeloma lesions. The recalcification was measured in both groups 4 and 12 weeks follow irradiation. Remineralization occurred in 32/101 patients (33.7%), of which 17 (53.2%) were complete and 15 (46.2%) partial. No differences in reossification were observed between 1 × 8 Gy vs. 10 × 3 Gy schedules [29].

The systematic review by Groenen estimated the impact of EBRT in stabilizing metastatic bone lesions, in particular the effect the radiotherapy alone and combined with bisphosphonates or RANK ligands (RANKL). The emphasis of this study was placed on biomechanical characteristics such as bone quality changes and pathological fractures. It was concluded that no sufficient evidence exist, which demonstrate the positive influence of the EBRT on bone density and pathological fracture respective [30]. The incidence of pathological fractures in all patients in our study at baseline and 6 months was moderate by 7.4 and 9% respectively [31]. None of the relevant studies provided information on the influence of fractionation on the stability of osseous metastases.

Despite the lack of evidence, our results confirm the clinical experience that radiotherapy is an effective local treatment for unstable SBM from solid tumors. To the best of our knowledge, this was the largest retrospective study to date investigating the influence of various multi-fraction palliative radiotherapy regimen on the stability of SBM. A consequence of this findings a prospective randomized study was initiated in our department to investigate the influence of different palliative multi-fraction schemata on the bone density of vertebral metastases [32].

In our cohort 62.6% of patients received antiresoptive treatment. In comparison with our results, Udagawa and co-worker reported higher rates of bone-target therapy with 69% (*n* = 103) of 149 patients with untreated bronchial carcinoma and osseous metastases [33]. Another study reported even higher rates with 89% in patients with advanced breast cancer and osseous metastases [34]. In group comparison, the prescription of antiresoptive treatment in our trial was significant higher with 66.7% vs 51.1% in the SCR group (*p* < 0.001). These findings may reflect a realization of palliative therapeutic approaches, especially rapid pain relief by shorter life expectancy. This difference in our study cannot be explained. Multicentric international comparisons of prescription patterns of antiresoptive treatment would be necessary.

Statistically, the median OS and BS was significantly better in the LCR group (9.5; 12.2 and 5.5; 6.7) months respectively, (log rank *p* < .0001). Stability did not impact OS in either group. Statistically, a significant percentage of patients was alive in LRT group at the time of the survey, which indirectly reflects the common practice, particularly the influence of subjective life expectancy estimation (KPS, extra osseous disease extent) on the choice of multi-fraction regime by radio-oncologists.

Although the strengths of our investigation include the representative large collective and standardized evaluation of bone density and stability of all osteolytic lesions, several limitations must be acknowledged. In addition to a retrospective character and the unequal number of patients in the comparative groups (SRT > LRT).

## Conclusion

Our study demonstrated no significant difference in recalcification rates between various multi-fraction schedules (SCR vs. LCR) in the palliative management of unstable SBM. Use of shorter, equally potent schemata may shorten hospitalization, increase the patients’ quality of life and save resources.

## Abbreviations

BS: Bone survival
CT: Computer tomography
CTV: Clinical target volume
EBRT: External beam radiotherapy
GTV: Gross tumor volume
Gy: Gray
KPS: Karnofsky performance status
LCR: Long course radiotherapy
OS: Overall survival
PTV: Planning target volume
RANKL: Receptor activator of nuclear factor kappa-B ligand
ROI: Region of interest
SBM: Spinal bone metastasis
SCR: Short course radiotherapy
